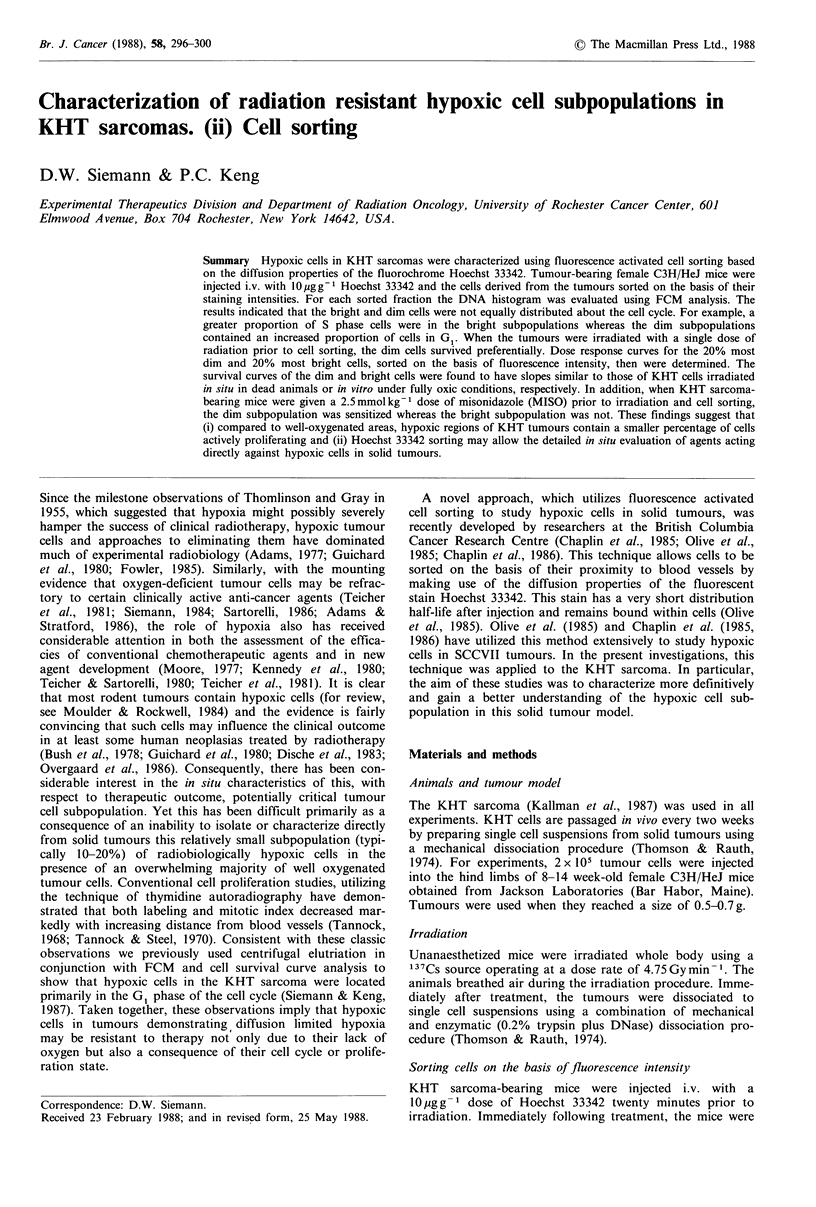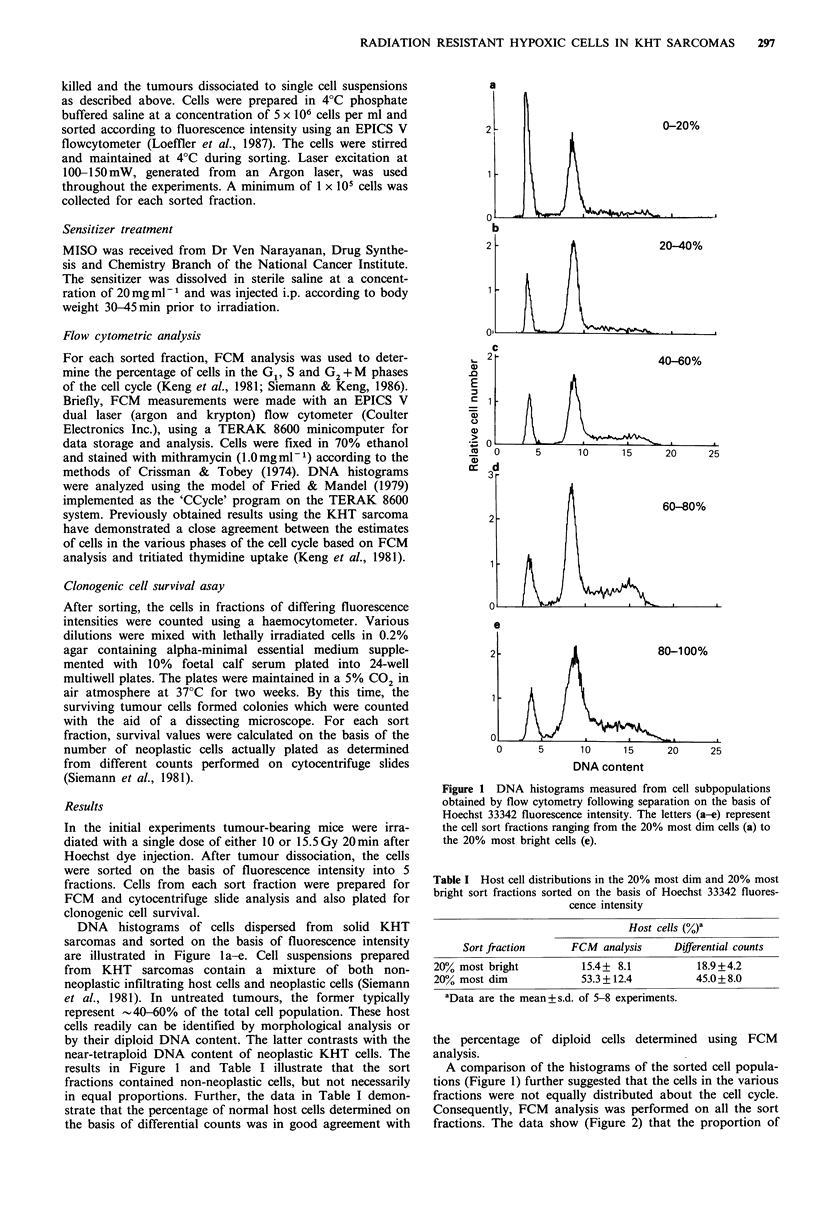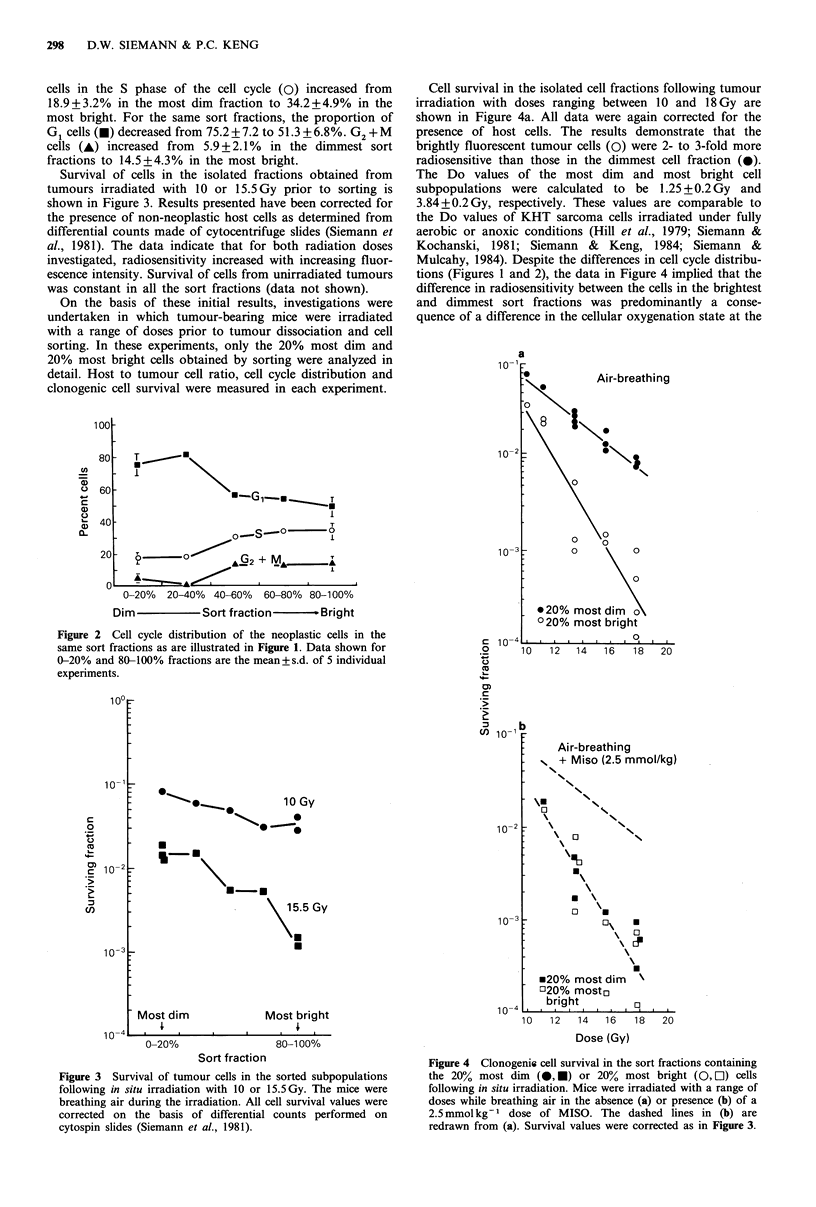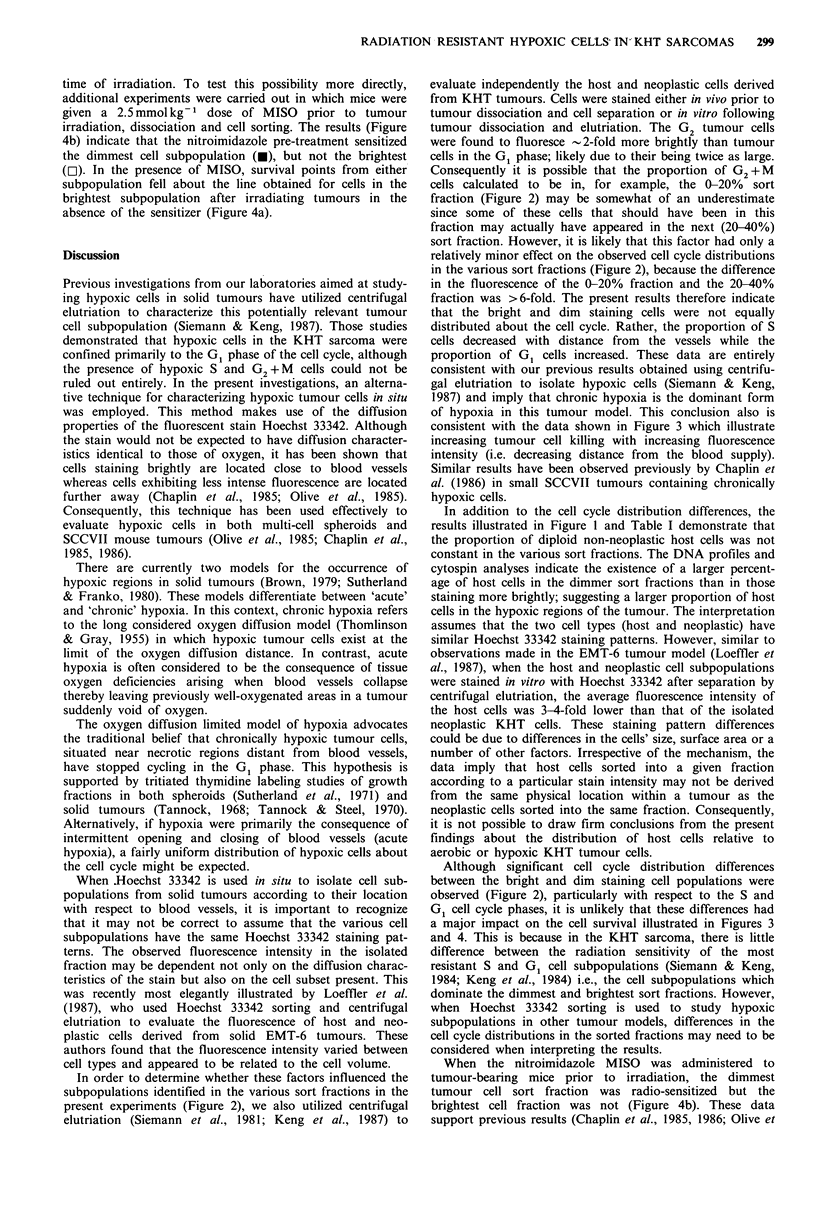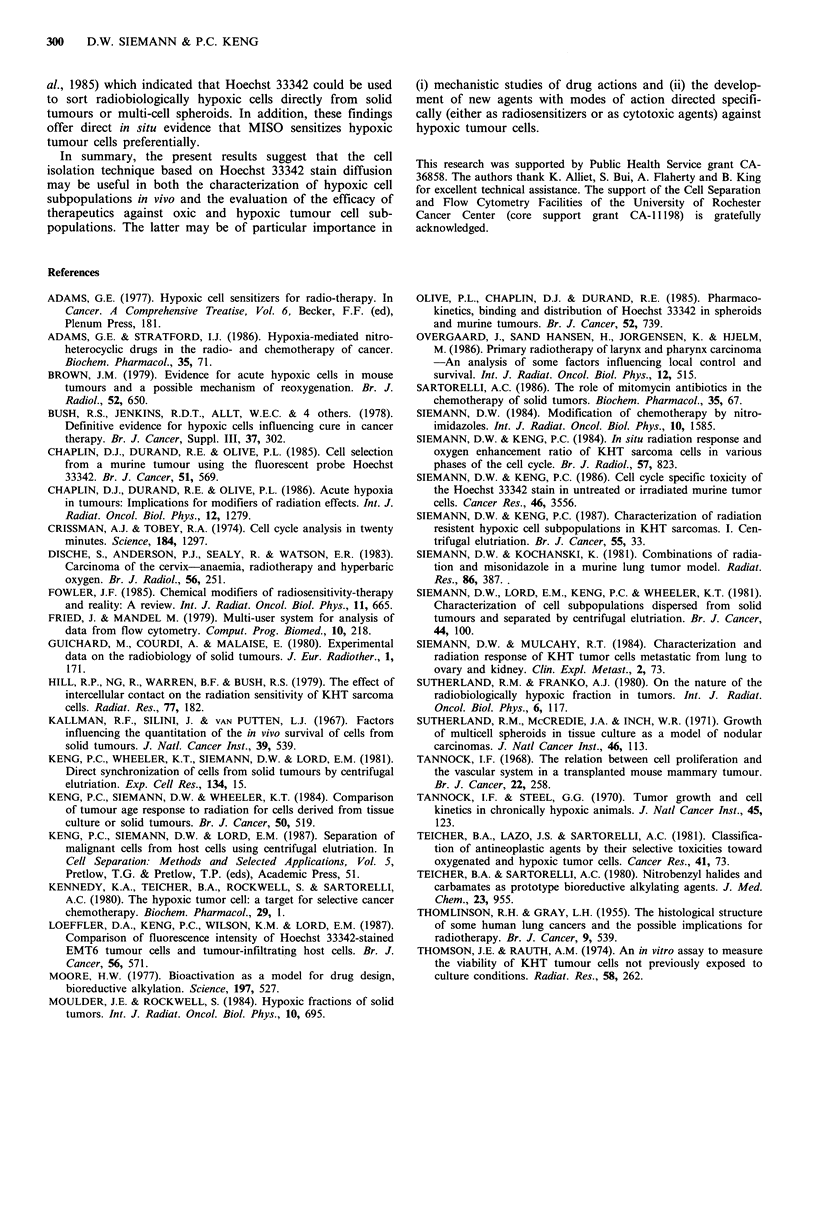# Characterization of radiation resistant hypoxic cell subpopulations in KHT sarcomas. (II). Cell sorting.

**DOI:** 10.1038/bjc.1988.207

**Published:** 1988-09

**Authors:** D. W. Siemann, P. C. Keng

**Affiliations:** Experimental Therapeutics Division, University of Rochester Cancer Center, New York 14642.

## Abstract

Hypoxic cells in KHT sarcomas were characterized using fluorescence activated cell sorting based on the diffusion properties of the fluorochrome Hoechst 33342. Tumour-bearing female C3H/HeJ mice were injected i.v. with 10 micrograms g-1 Hoechst 33342 and the cells derived from the tumours sorted on the basis of their staining intensities. For each sorted fraction the DNA histogram was evaluated using FCM analysis. The results indicated that the bright and dim cells were not equally distributed about the cell cycle. For example, a greater proportion of S phase cells were in the bright subpopulations whereas the dim subpopulations contained an increased proportion of cells in G1. When the tumours were irradiated with a single dose of radiation prior to cell sorting, the dim cells survived preferentially. Dose response curves for the 20% most dim and 20% most bright cells, sorted on the basis of fluorescence intensity, then were determined. The survival curves of the dim and bright cells were found to have slopes similar to those of KHT cells irradiated in situ in dead animals or in vitro under fully oxic conditions, respectively. In addition, when KHT sarcoma-bearing mice were given a 2.5 mmol kg-1 dose of misonidazole (MISO) prior to irradiation and cell sorting, the dim subpopulation was sensitized whereas the bright subpopulation was not. These findings suggest that (i) compared to well-oxygenated areas, hypoxic regions of KHT tumours contain a smaller percentage of cells actively proliferating and (ii) Hoechst 33342 sorting may allow the detailed in situ evaluation of agents acting directly against hypoxic cells in solid tumours.


Br. J. Cancer (1988), 58, 296–300 © The Macmillan Press Ltd., 1988

### Characterization of radiation resistant hypoxic cell subpopulations in KHT sarcomas. (ii) Cell sorting

D.W. Siemann & P.C. Keng

*Experimental Therapeutics Division and Department of Radiation Oncology, University of Rochester Cancer Center, 601 Elmwood Avenue, Box 704 Rochester, New York 14642, USA.*

**Summary** Hypoxic cells in KHT sarcomas were characterized using fluorescence activated cell sorting based on the diffusion properties of the fluorochrome Hoechst 33342. Tumour-bearing female C3H/HeJ mice were injected i.v. with $10\,\mu g\,g^{-1}$ Hoechst 33342 and the cells derived from the tumours sorted on the basis of their staining intensities. For each sorted fraction the DNA histogram was evaluated using FCM analysis. The results indicated that the bright and dim cells were not equally distributed about the cell cycle. For example, a greater proportion of S phase cells were in the bright subpopulations whereas the dim subpopulations contained an increased proportion of cells in $G_1$. When the tumours were irradiated with a single dose of radiation prior to cell sorting, the dim cells survived preferentially. Dose response curves for the 20% most dim and 20% most bright cells, sorted on the basis of fluorescence intensity, then were determined. The survival curves of the dim and bright cells were found to have slopes similar to those of KHT cells irradiated *in situ* in dead animals or *in vitro* under fully oxic conditions, respectively. In addition, when KHT sarcoma-bearing mice were given a $2.5\,mmol\,kg^{-1}$ dose of misonidazole (MISO) prior to irradiation and cell sorting, the dim subpopulation was sensitized whereas the bright subpopulation was not. These findings suggest that (i) compared to well-oxygenated areas, hypoxic regions of KHT tumours contain a smaller percentage of cells actively proliferating and (ii) Hoechst 33342 sorting may allow the detailed *in situ* evaluation of agents acting directly against hypoxic cells in solid tumours.

Since the milestone observations of Thomlinson and Gray in 1955, which suggested that hypoxia might possibly severely hamper the success of clinical radiotherapy, hypoxic tumour cells and approaches to eliminating them have dominated much of experimental radiobiology (Adams, 1977; Guichard *et al.*, 1980; Fowler, 1985). Similarly, with the mounting evidence that oxygen-deficient tumour cells may be refractory to certain clinically active anti-cancer agents (Teicher *et al.*, 1981; Siemann, 1984; Sartorelli, 1986; Adams & Stratford, 1986), the role of hypoxia also has received considerable attention in both the assessment of the efficacies of conventional chemotherapeutic agents and in new agent development (Moore, 1977; Kennedy *et al.*, 1980; Teicher & Sartorelli, 1980; Teicher *et al.*, 1981). It is clear that most rodent tumours contain hypoxic cells (for review, see Moulder & Rockwell, 1984) and the evidence is fairly convincing that such cells may influence the clinical outcome in at least some human neoplasias treated by radiotherapy (Bush *et al.*, 1978; Guichard *et al.*, 1980; Dische *et al.*, 1983; Overgaard *et al.*, 1986). Consequently, there has been considerable interest in the *in situ* characteristics of this, with respect to therapeutic outcome, potentially critical tumour cell subpopulation. Yet this has been difficult primarily as a consequence of an inability to isolate or characterize directly from solid tumours this relatively small subpopulation (typically 10–20%) of radiobiologically hypoxic cells in the presence of an overwhelming majority of well oxygenated tumour cells. Conventional cell proliferation studies, utilizing the technique of thymidine autoradiography have demonstrated that both labeling and mitotic index decreased markedly with increasing distance from blood vessels (Tannock, 1968; Tannock & Steel, 1970). Consistent with these classic observations we previously used centrifugal elutriation in conjunction with FCM and cell survival curve analysis to show that hypoxic cells in the KHT sarcoma were located primarily in the $G_1$ phase of the cell cycle (Siemann & Keng, 1987). Taken together, these observations imply that hypoxic cells in tumours demonstrating diffusion limited hypoxia may be resistant to therapy not only due to their lack of oxygen but also a consequence of their cell cycle or proliferation state.

Correspondence: D.W. Siemann.
Received 23 February 1988; and in revised form, 25 May 1988.

A novel approach, which utilizes fluorescence activated cell sorting to study hypoxic cells in solid tumours, was recently developed by researchers at the British Columbia Cancer Research Centre (Chaplin *et al.*, 1985; Olive *et al.*, 1985; Chaplin *et al.*, 1986). This technique allows cells to be sorted on the basis of their proximity to blood vessels by making use of the diffusion properties of the fluorescent stain Hoechst 33342. This stain has a very short distribution half-life after injection and remains bound within cells (Olive *et al.*, 1985). Olive *et al.* (1985) and Chaplin *et al.* (1985, 1986) have utilized this method extensively to study hypoxic cells in SCCVII tumours. In the present investigations, this technique was applied to the KHT sarcoma. In particular, the aim of these studies was to characterize more definitively and gain a better understanding of the hypoxic cell subpopulation in this solid tumour model.

### Materials and methods

### *Animals and tumour model*

The KHT sarcoma (Kallman *et al.*, 1987) was used in all experiments. KHT cells are passaged *in vivo* every two weeks by preparing single cell suspensions from solid tumours using a mechanical dissociation procedure (Thomson & Rauth, 1974). For experiments, $2\times10^5$ tumour cells were injected into the hind limbs of 8–14 week-old female C3H/HeJ mice obtained from Jackson Laboratories (Bar Habor, Maine). Tumours were used when they reached a size of 0.5–0.7 g.

### *Irradiation*

Unanaesthetized mice were irradiated whole body using a $^{137}Cs$ source operating at a dose rate of $4.75\,Gy\,min^{-1}$. The animals breathed air during the irradiation procedure. Immediately after treatment, the tumours were dissociated to single cell suspensions using a combination of mechanical and enzymatic (0.2% trypsin plus DNase) dissociation procedure (Thomson & Rauth, 1974).

### *Sorting cells on the basis of fluorescence intensity*

KHT sarcoma-bearing mice were injected i.v. with a $10\,\mu g\,g^{-1}$ dose of Hoechst 33342 twenty minutes prior to irradiation. Immediately following treatment, the mice were killed and the tumours dissociated to single cell suspensions as described above. Cells were prepared in 4°C phosphate buffered saline at a concentration of $5 \times 10^6$ cells per ml and sorted according to fluorescence intensity using an EPICS V flowcytometer (Loeffler *et al.*, 1987). The cells were stirred and maintained at 4°C during sorting. Laser excitation at 100–150 mW, generated from an Argon laser, was used throughout the experiments. A minimum of $1 \times 10^5$ cells was collected for each sorted fraction.

*Sensitizer treatment*

MISO was received from Dr Ven Narayanan, Drug Synthesis and Chemistry Branch of the National Cancer Institute. The sensitizer was dissolved in sterile saline at a concentration of 20 mg ml$^{-1}$ and was injected i.p. according to body weight 30–45 min prior to irradiation.

*Flow cytometric analysis*

For each sorted fraction, FCM analysis was used to determine the percentage of cells in the $G_1$, S and $G_2$+M phases of the cell cycle (Keng *et al.*, 1981; Siemann & Keng, 1986). Briefly, FCM measurements were made with an EPICS V dual laser (argon and krypton) flow cytometer (Coulter Electronics Inc.), using a TERAK 8600 minicomputer for data storage and analysis. Cells were fixed in 70% ethanol and stained with mithramycin (1.0 mg ml$^{-1}$) according to the methods of Crissman & Tobey (1974). DNA histograms were analyzed using the model of Fried & Mandel (1979) implemented as the 'CCycle' program on the TERAK 8600 system. Previously obtained results using the KHT sarcoma have demonstrated a close agreement between the estimates of cells in the various phases of the cell cycle based on FCM analysis and tritiated thymidine uptake (Keng *et al.*, 1981).

*Clonogenic cell survival asay*

After sorting, the cells in fractions of differing fluorescence intensities were counted using a haemocytometer. Various dilutions were mixed with lethally irradiated cells in 0.2% agar containing alpha-minimal essential medium supplemented with 10% foetal calf serum plated into 24-well multiwell plates. The plates were maintained in a 5% $CO_2$ in air atmosphere at 37°C for two weeks. By this time, the surviving tumour cells formed colonies which were counted with the aid of a dissecting microscope. For each sort fraction, survival values were calculated on the basis of the number of neoplastic cells actually plated as determined from different counts performed on cytocentrifuge slides (Siemann *et al.*, 1981).

*Results*

In the initial experiments tumour-bearing mice were irradiated with a single dose of either 10 or 15.5 Gy 20 min after Hoechst dye injection. After tumour dissociation, the cells were sorted on the basis of fluorescence intensity into 5 fractions. Cells from each sort fraction were prepared for FCM and cytocentrifuge slide analysis and also plated for clonogenic cell survival.

DNA histograms of cells dispersed from solid KHT sarcomas and sorted on the basis of fluorescence intensity are illustrated in Figure 1a–e. Cell suspensions prepared from KHT sarcomas contain a mixture of both non-neoplastic infiltrating host cells and neoplastic cells (Siemann *et al.*, 1981). In untreated tumours, the former typically represent ~40–60% of the total cell population. These host cells readily can be identified by morphological analysis or by their diploid DNA content. The latter contrasts with the near-tetraploid DNA content of neoplastic KHT cells. The results in Figure 1 and Table I illustrate that the sort fractions contained non-neoplastic cells, but not necessarily in equal proportions. Further, the data in Table I demonstrate that the percentage of normal host cells determined on the basis of differential counts was in good agreement with the percentage of diploid cells determined using FCM analysis.

A comparison of the histograms of the sorted cell populations (Figure 1) further suggested that the cells in the various fractions were not equally distributed about the cell cycle. Consequently, FCM analysis was performed on all the sort fractions. The data show (Figure 2) that the proportion of

**Figure 1** DNA histograms measured from cell subpopulations obtained by flow cytometry following separation on the basis of Hoechst 33342 fluorescence intensity. The letters (**a**–**e**) represent the cell sort fractions ranging from the 20% most dim cells (**a**) to the 20% most bright cells (**e**).

**Table I** Host cell distributions in the 20% most dim and 20% most bright sort fractions sorted on the basis of Hoechst 33342 fluorescence intensity

| | Host cells (%)[a] | |
|---|---|---|
| *Sort fraction* | *FCM analysis* | *Differential counts* |
| 20% most bright | 15.4± 8.1 | 18.9±4.2 |
| 20% most dim | 53.3±12.4 | 45.0±8.0 |

[a]Data are the mean±s.d. of 5–8 experiments.

cells in the S phase of the cell cycle (○) increased from 18.9±3.2% in the most dim fraction to 34.2±4.9% in the most bright. For the same sort fractions, the proportion of $G_1$ cells (■) decreased from 75.2±7.2 to 51.3±6.8%. $G_2$+M cells (▲) increased from 5.9±2.1% in the dimmest sort fractions to 14.5±4.3% in the most bright.

Survival of cells in the isolated fractions obtained from tumours irradiated with 10 or 15.5 Gy prior to sorting is shown in Figure 3. Results presented have been corrected for the presence of non-neoplastic host cells as determined from differential counts made of cytocentrifuge slides (Siemann *et al.*, 1981). The data indicate that for both radiation doses investigated, radiosensitivity increased with increasing fluorescence intensity. Survival of cells from unirradiated tumours was constant in all the sort fractions (data not shown).

On the basis of these initial results, investigations were undertaken in which tumour-bearing mice were irradiated with a range of doses prior to tumour dissociation and cell sorting. In these experiments, only the 20% most dim and 20% most bright cells obtained by sorting were analyzed in detail. Host to tumour cell ratio, cell cycle distribution and clonogenic cell survival were measured in each experiment.

**Figure 2** Cell cycle distribution of the neoplastic cells in the same sort fractions as are illustrated in **Figure 1**. Data shown for 0–20% and 80–100% fractions are the mean±s.d. of 5 individual experiments.

**Figure 3** Survival of tumour cells in the sorted subpopulations following *in situ* irradiation with 10 or 15.5 Gy. The mice were breathing air during the irradiation. All cell survival values were corrected on the basis of differential counts performed on cytospin slides (Siemann *et al.*, 1981).

Cell survival in the isolated cell fractions following tumour irradiation with doses ranging between 10 and 18 Gy are shown in Figure 4a. All data were again corrected for the presence of host cells. The results demonstrate that the brightly fluorescent tumour cells (○) were 2- to 3-fold more radiosensitive than those in the dimmest cell fraction (●). The Do values of the most dim and most bright cell subpopulations were calculated to be 1.25±0.2 Gy and 3.84±0.2 Gy, respectively. These values are comparable to the Do values of KHT sarcoma cells irradiated under fully aerobic or anoxic conditions (Hill *et al.*, 1979; Siemann & Kochanski, 1981; Siemann & Keng, 1984; Siemann & Mulcahy, 1984). Despite the differences in cell cycle distributions (Figures 1 and 2), the data in Figure 4 implied that the difference in radiosensitivity between the cells in the brightest and dimmest sort fractions was predominantly a consequence of a difference in the cellular oxygenation state at the

**Figure 4** Clonogenic cell survival in the sort fractions containing the 20% most dim (●, ■) or 20% most bright (○, □) cells following *in situ* irradiation. Mice were irradiated with a range of doses while breathing air in the absence (**a**) or presence (**b**) of a 2.5 mmol $kg^{-1}$ dose of MISO. The dashed lines in (**b**) are redrawn from (**a**). Survival values were corrected as in **Figure 3**.

time of irradiation. To test this possibility more directly, additional experiments were carried out in which mice were given a $2.5\,mmol\,kg^{-1}$ dose of MISO prior to tumour irradiation, dissociation and cell sorting. The results (Figure 4b) indicate that the nitroimidazole pre-treatment sensitized the dimmest cell subpopulation (■), but not the brightest (□). In the presence of MISO, survival points from either subpopulation fell about the line obtained for cells in the brightest subpopulation after irradiating tumours in the absence of the sensitizer (Figure 4a).

### Discussion

Previous investigations from our laboratories aimed at studying hypoxic cells in solid tumours have utilized centrifugal elutriation to characterize this potentially relevant tumour cell subpopulation (Siemann & Keng, 1987). Those studies demonstrated that hypoxic cells in the KHT sarcoma were confined primarily to the $G_1$ phase of the cell cycle, although the presence of hypoxic S and $G_2+M$ cells could not be ruled out entirely. In the present investigations, an alternative technique for characterizing hypoxic tumour cells *in situ* was employed. This method makes use of the diffusion properties of the fluorescent stain Hoechst 33342. Although the stain would not be expected to have diffusion characteristics identical to those of oxygen, it has been shown that cells staining brightly are located close to blood vessels whereas cells exhibiting less intense fluorescence are located further away (Chaplin *et al.*, 1985; Olive *et al.*, 1985). Consequently, this technique has been used effectively to evaluate hypoxic cells in both multi-cell spheroids and SCCVII mouse tumours (Olive *et al.*, 1985; Chaplin *et al.*, 1985, 1986).

There are currently two models for the occurrence of hypoxic regions in solid tumours (Brown, 1979; Sutherland & Franko, 1980). These models differentiate between 'acute' and 'chronic' hypoxia. In this context, chronic hypoxia refers to the long considered oxygen diffusion model (Thomlinson & Gray, 1955) in which hypoxic tumour cells exist at the limit of the oxygen diffusion distance. In contrast, acute hypoxia is often considered to be the consequence of tissue oxygen deficiencies arising when blood vessels collapse thereby leaving previously well-oxygenated areas in a tumour suddenly void of oxygen.

The oxygen diffusion limited model of hypoxia advocates the traditional belief that chronically hypoxic tumour cells, situated near necrotic regions distant from blood vessels, have stopped cycling in the $G_1$ phase. This hypothesis is supported by tritiated thymidine labeling studies of growth fractions in both spheroids (Sutherland *et al.*, 1971) and solid tumours (Tannock, 1968; Tannock & Steel, 1970). Alternatively, if hypoxia were primarily the consequence of intermittent opening and closing of blood vessels (acute hypoxia), a fairly uniform distribution of hypoxic cells about the cell cycle might be expected.

When Hoechst 33342 is used *in situ* to isolate cell subpopulations from solid tumours according to their location with respect to blood vessels, it is important to recognize that it may not be correct to assume that the various cell subpopulations have the same Hoechst 33342 staining patterns. The observed fluorescence intensity in the isolated fraction may be dependent not only on the diffusion characteristics of the stain but also on the cell subset present. This was recently most elegantly illustrated by Loeffler *et al.* (1987), who used Hoechst 33342 sorting and centrifugal elutriation to evaluate the fluorescence of host and neoplastic cells derived from solid EMT-6 tumours. These authors found that the fluorescence intensity varied between cell types and appeared to be related to the cell volume.

In order to determine whether these factors influenced the subpopulations identified in the various sort fractions in the present experiments (Figure 2), we also utilized centrifugal elutriation (Siemann *et al.*, 1981; Keng *et al.*, 1987) to evaluate independently the host and neoplastic cells derived from KHT tumours. Cells were stained either *in vivo* prior to tumour dissociation and cell separation or *in vitro* following tumour dissociation and elutriation. The $G_2$ tumour cells were found to fluoresce ~2-fold more brightly than tumour cells in the $G_1$ phase; likely due to their being twice as large. Consequently it is possible that the proportion of $G_2+M$ cells calculated to be in, for example, the 0–20% sort fraction (Figure 2) may be somewhat of an underestimate since some of these cells that should have been in this fraction may actually have appeared in the next (20–40%) sort fraction. However, it is likely that this factor had only a relatively minor effect on the observed cell cycle distributions in the various sort fractions (Figure 2), because the difference in the fluorescence of the 0–20% fraction and the 20–40% fraction was >6-fold. The present results therefore indicate that the bright and dim staining cells were not equally distributed about the cell cycle. Rather, the proportion of S cells decreased with distance from the vessels while the proportion of $G_1$ cells increased. These data are entirely consistent with our previous results obtained using centrifugal elutriation to isolate hypoxic cells (Siemann & Keng, 1987) and imply that chronic hypoxia is the dominant form of hypoxia in this tumour model. This conclusion also is consistent with the data shown in Figure 3 which illustrate increasing tumour cell killing with increasing fluorescence intensity (i.e. decreasing distance from the blood supply). Similar results have been observed previously by Chaplin *et al.* (1986) in small SCCVII tumours containing chronically hypoxic cells.

In addition to the cell cycle distribution differences, the results illustrated in Figure 1 and Table I demonstrate that the proportion of diploid non-neoplastic host cells was not constant in the various sort fractions. The DNA profiles and cytospin analyses indicate the existence of a larger percentage of host cells in the dimmer sort fractions than in those staining more brightly; suggesting a larger proportion of host cells in the hypoxic regions of the tumour. The interpretation assumes that the two cell types (host and neoplastic) have similar Hoechst 33342 staining patterns. However, similar to observations made in the EMT-6 tumour model (Loeffler *et al.*, 1987), when the host and neoplastic cell subpopulations were stained *in vitro* with Hoechst 33342 after separation by centrifugal elutriation, the average fluorescence intensity of the host cells was 3–4-fold lower than that of the isolated neoplastic KHT cells. These staining pattern differences could be due to differences in the cells' size, surface area or a number of other factors. Irrespective of the mechanism, the data imply that host cells sorted into a given fraction according to a particular stain intensity may not be derived from the same physical location within a tumour as the neoplastic cells sorted into the same fraction. Consequently, it is not possible to draw firm conclusions from the present findings about the distribution of host cells relative to aerobic or hypoxic KHT tumour cells.

Although significant cell cycle distribution differences between the bright and dim staining cell populations were observed (Figure 2), particularly with respect to the S and $G_1$ cell cycle phases, it is unlikely that these differences had a major impact on the cell survival illustrated in Figures 3 and 4. This is because in the KHT sarcoma, there is little difference between the radiation sensitivity of the most resistant S and $G_1$ cell subpopulations (Siemann & Keng, 1984; Keng *et al.*, 1984) i.e., the cell subpopulations which dominate the dimmest and brightest sort fractions. However, when Hoechst 33342 sorting is used to study hypoxic subpopulations in other tumour models, differences in the cell cycle distributions in the sorted fractions may need to be considered when interpreting the results.

When the nitroimidazole MISO was administered to tumour-bearing mice prior to irradiation, the dimmest tumour cell sort fraction was radio-sensitized but the brightest cell fraction was not (Figure 4b). These data support previous results (Chaplin *et al.*, 1985, 1986; Olive *et*

*al.*, 1985) which indicated that Hoechst 33342 could be used to sort radiobiologically hypoxic cells directly from solid tumours or multi-cell spheroids. In addition, these findings offer direct *in situ* evidence that MISO sensitizes hypoxic tumour cells preferentially.

In summary, the present results suggest that the cell isolation technique based on Hoechst 33342 stain diffusion may be useful in both the characterization of hypoxic cell subpopulations *in vivo* and the evaluation of the efficacy of therapeutics against oxic and hypoxic tumour cell subpopulations. The latter may be of particular importance in (i) mechanistic studies of drug actions and (ii) the development of new agents with modes of action directed specifically (either as radiosensitizers or as cytotoxic agents) against hypoxic tumour cells.

This research was supported by Public Health Service grant CA-36858. The authors thank K. Alliet, S. Bui, A. Flaherty and B. King for excellent technical assistance. The support of the Cell Separation and Flow Cytometry Facilities of the University of Rochester Cancer Center (core support grant CA-11198) is gratefully acknowledged.

### References

ADAMS, G.E. (1977). Hypoxic cell sensitizers for radio-therapy. In *Cancer. A Comprehensive Treatise, Vol. 6*, Becker, F.F. (ed), Plenum Press, 181.

ADAMS, G.E. & STRATFORD, I.J. (1986). Hypoxia-mediated nitro-heterocyclic drugs in the radio- and chemotherapy of cancer. *Biochem. Pharmacol.*, **35**, 71.

BROWN, J.M. (1979). Evidence for acute hypoxic cells in mouse tumours and a possible mechanism of reoxygenation. *Br. J. Radiol.*, **52**, 650.

BUSH, R.S., JENKINS, R.D.T., ALLT, W.E.C. & 4 others. (1978). Definitive evidence for hypoxic cells influencing cure in cancer therapy. *Br. J. Cancer*, Suppl. III, **37**, 302.

CHAPLIN, D.J., DURAND, R.E. & OLIVE, P.L. (1985). Cell selection from a murine tumour using the fluorescent probe Hoechst 33342. *Br. J. Cancer*, **51**, 569.

CHAPLIN, D.J., DURAND, R.E. & OLIVE, P.L. (1986). Acute hypoxia in tumours: Implications for modifiers of radiation effects. *Int. J. Radiat. Oncol. Biol. Phys.*, **12**, 1279.

CRISSMAN, A.J. & TOBEY, R.A. (1974). Cell cycle analysis in twenty minutes. *Science*, **184**, 1297.

DISCHE, S., ANDERSON, P.J., SEALY, R. & WATSON, E.R. (1983). Carcinoma of the cervix—anaemia, radiotherapy and hyperbaric oxygen. *Br. J. Radiol.*, **56**, 251.

FOWLER, J.F. (1985). Chemical modifiers of radiosensitivity-therapy and reality: A review. *Int. J. Radiat. Oncol. Biol. Phys.*, **11**, 665.

FRIED, J. & MANDEL M. (1979). Multi-user system for analysis of data from flow cytometry. *Comput. Prog. Biomed.*, **10**, 218.

GUICHARD, M., COURDI, A. & MALAISE, E. (1980). Experimental data on the radiobiology of solid tumours. *J. Eur. Radiother.*, **1**, 171.

HILL, R.P., NG, R., WARREN, B.F. & BUSH, R.S. (1979). The effect of intercellular contact on the radiation sensitivity of KHT sarcoma cells. *Radiat. Res.*, **77**, 182.

KALLMAN, R.F., SILINI, J. & VAN PUTTEN, L.J. (1967). Factors influencing the quantitation of the *in vivo* survival of cells from solid tumours. *J. Natl. Cancer Inst.*, **39**, 539.

KENG, P.C., WHEELER, K.T., SIEMANN, D.W. & LORD, E.M. (1981). Direct synchronization of cells from solid tumours by centrifugal elutriation. *Exp. Cell Res.*, **134**, 15.

KENG, P.C., SIEMANN, D.W. & WHEELER, K.T. (1984). Comparison of tumour age response to radiation for cells derived from tissue culture or solid tumours. *Br. J. Cancer*, **50**, 519.

KENG, P.C., SIEMANN, D.W. & LORD, E.M. (1987). Separation of malignant cells from host cells using centrifugal elutriation. In *Cell Separation: Methods and Selected Applications, Vol. 5*, Pretlow, T.G. & Pretlow, T.P. (eds), Academic Press, 51.

KENNEDY, K.A., TEICHER, B.A., ROCKWELL, S. & SARTORELLI, A.C. (1980). The hypoxic tumor cell: a target for selective cancer chemotherapy. *Biochem. Pharmacol.*, **29**, 1.

LOEFFLER, D.A., KENG, P.C., WILSON, K.M. & LORD, E.M. (1987). Comparison of fluorescence intensity of Hoechst 33342-stained EMT6 tumour cells and tumour-infiltrating host cells. *Br. J. Cancer*, **56**, 571.

MOORE, H.W. (1977). Bioactivation as a model for drug design, bioreductive alkylation. *Science*, **197**, 527.

MOULDER, J.E. & ROCKWELL, S. (1984). Hypoxic fractions of solid tumors. *Int. J. Radiat. Oncol. Biol. Phys.*, **10**, 695.

OLIVE, P.L., CHAPLIN, D.J. & DURAND, R.E. (1985). Pharmacokinetics, binding and distribution of Hoechst 33342 in spheroids and murine tumours. *Br. J. Cancer*, **52**, 739.

OVERGAARD, J., SAND HANSEN, H., JORGENSEN, K. & HJELM, M. (1986). Primary radiotherapy of larynx and pharynx carcinoma —An analysis of some factors influencing local control and survival. *Int. J. Radiat. Oncol. Biol. Phys.*, **12**, 515.

SARTORELLI, A.C. (1986). The role of mitomycin antibiotics in the chemotherapy of solid tumors. *Biochem. Pharmacol.*, **35**, 67.

SIEMANN, D.W. (1984). Modification of chemotherapy by nitro-imidazoles. *Int. J. Radiat. Oncol. Biol. Phys.*, **10**, 1585.

SIEMANN, D.W. & KENG, P.C. (1984). *In situ* radiation response and oxygen enhancement ratio of KHT sarcoma cells in various phases of the cell cycle. *Br. J. Radiol.*, **57**, 823.

SIEMANN, D.W. & KENG, P.C. (1986). Cell cycle specific toxicity of the Hoechst 33342 stain in untreated or irradiated murine tumor cells. *Cancer Res.*, **46**, 3556.

SIEMANN, D.W. & KENG, P.C. (1987). Characterization of radiation resistent hypoxic cell subpopulations in KHT sarcomas. I. Centrifugal elutriation. *Br. J. Cancer*, **55**, 33.

SIEMANN, D.W. & KOCHANSKI, K. (1981). Combinations of radiation and misonidazole in a murine lung tumor model. *Radiat. Res.*, **86**, 387. .

SIEMANN, D.W., LORD, E.M., KENG, P.C. & WHEELER, K.T. (1981). Characterization of cell subpopulations dispersed from solid tumours and separated by centrifugal elutriation. *Br. J. Cancer*, **44**, 100.

SIEMANN, D.W. & MULCAHY, R.T. (1984). Characterization and radiation response of KHT tumor cells metastatic from lung to ovary and kidney. *Clin. Expl. Metast.*, **2**, 73.

SUTHERLAND, R.M. & FRANKO, A.J. (1980). On the nature of the radiobiologically hypoxic fraction in tumors. *Int. J. Radiat. Oncol. Biol. Phys.*, **6**, 117.

SUTHERLAND, R.M., MCCREDIE, J.A. & INCH, W.R. (1971). Growth of multicell spheroids in tissue culture as a model of nodular carcinomas. *J. Natl Cancer Inst.*, **46**, 113.

TANNOCK, I.F. (1968). The relation between cell proliferation and the vascular system in a transplanted mouse mammary tumour. *Br. J. Cancer*, **22**, 258.

TANNOCK, I.F. & STEEL, G.G. (1970). Tumor growth and cell kinetics in chronically hypoxic animals. *J. Natl Cancer Inst.*, **45**, 123.

TEICHER, B.A., LAZO, J.S. & SARTORELLI, A.C. (1981). Classification of antineoplastic agents by their selective toxicities toward oxygenated and hypoxic tumor cells. *Cancer Res.*, **41**, 73.

TEICHER, B.A. & SARTORELLI, A.C. (1980). Nitrobenzyl halides and carbamates as prototype bioreductive alkylating agents. *J. Med. Chem.*, **23**, 955.

THOMLINSON, R.H. & GRAY, L.H. (1955). The histological structure of some human lung cancers and the possible implications for radiotherapy. *Br. J. Cancer*, **9**, 539.

THOMSON, J.E. & RAUTH, A.M. (1974). An *in vitro* assay to measure the viability of KHT tumour cells not previously exposed to culture conditions. *Radiat. Res.*, **58**, 262.